# 26 Kawasaki’s disease vs systemic juvenile idiopathic arthritis, a diagnostic dilemma

**DOI:** 10.1093/rheumatology/keac496.022

**Published:** 2022-10-07

**Authors:** Monali Thakkar, Angela Migowa

**Affiliations:** Aga Khan University Nairobi

## Abstract

**Background:**

Both these conditions are separate inflammatory conditions whose clinical presentation overlap and thus make their distinction difficult. Each has a criterion that allows diagnosis but since features overlap, diagnoses may be missed.

Recent research also shows that both disease processes have similar underlying pathogenesis and involve interleukin 1; which have now raised the question of whether both these disease processes are distinct entities or conditions of a similar spectrum.

**Methods:**

Medical records of from the inpatient admission at current and referring hospitals which included clinical history and examination, laboratory and radiological findings.

**Results:**

13-year-old boy referred from Isiolo county with complains of unremitting fevers for 9 days with associated hip pains. Was initially treated for malaria, and started on broad spectrum antibiotics and antivirals. On further examination, was noted to have cracked lips, strawberry tongue, non-suppurative conjuctivitis and an anterior chest wall wheal like rash. Examination also revealed a left sided non tender submandibular lymph node. Initial laboratory investigations showed neutrophilia of 12.89, thrombocytosis of 431, CRP of 364 and normal joint radiographs. An ECHO done showed left ventricular enlargement with corresponding mild Mitral regurgitation. Blood cultures showed no organisms. Autoimmune markers and tropical fever markers were normal as well. He was then placed on prednisolone challenge and reported to be fever free for the first time in 12 days.

**Discussion and conclusion:**

A persistent fever unremitting with parenteral antipyretics can have a multitude of differentials. In a tropical nation the likelihood of an infectious cause is more common than other aetiologies. Hence, it should always be investigated for thoroughly. In the case highlighted above, malaria, TB, osteomyelitis, septic arthritis and other infectious causes were investigated. Childhood malignancy can also present with the same. But of note is autoimmune conditions need to be considered. With history of arthritic like pain, quotidian pattern of fevers, evanescent salmon coloured chest wall rash and cervical lymphadenopathy, a diagnosis of Systemic Juvenile Arthritis needs to be considered. Often this may be overlooked or misinterpreted as Kawasaki’s; which is a close differential; or Still’s disease. There is a criterion for SJIA by the International League of Associations for Rheumatology, however, there can be an incomplete presentation as well. This adds on to the diagnostic challenge and hence, SJIA may remain undiagnosed for a long time.

